# Triclocarban Mediates Induction of Xenobiotic Metabolism through Activation of the Constitutive Androstane Receptor and the Estrogen Receptor Alpha

**DOI:** 10.1371/journal.pone.0037705

**Published:** 2012-06-15

**Authors:** Mei-Fei Yueh, Tao Li, Ronald M. Evans, Bruce Hammock, Robert H. Tukey

**Affiliations:** 1 Laboratory of Environmental Toxicology, Department of Pharmacology, University of California San Diego, La Jolla, California, United States of America; 2 Laboratory of Environmental Toxicology, Department of Chemistry and Biochemistry, University of California San Diego, La Jolla, California, United States of America; 3 Gene Expression Laboratory, Howard Hughes Medical Institute, The Salk Institute for Biological Studies, La Jolla, California, United States of America; 4 Department of Entomology University of California Davis Cancer Center, University of California Davis, Davis California, United States of America; Laurentian University, Canada

## Abstract

Triclocarban (3,4,4′-trichlorocarbanilide, TCC) is used as a broad-based antimicrobial agent that is commonly added to personal hygiene products. Because of its extensive use in the health care industry and resistance to degradation in sewage treatment processes, TCC has become a significant waste product that is found in numerous environmental compartments where humans and wildlife can be exposed. While TCC has been linked to a range of health and environmental effects, few studies have been conducted linking exposure to TCC and induction of xenobiotic metabolism through regulation by environmental sensors such as the nuclear xenobiotic receptors (XenoRs). To identify the ability of TCC to activate xenobiotic sensors, we monitored XenoR activities in response to TCC treatment using luciferase-based reporter assays. Among the XenoRs in the reporter screening assay, TCC promotes both constitutive androstane receptor (CAR) and estrogen receptor alpha (ERα) activities. TCC treatment to *hUGT1* mice resulted in induction of the *UGT1A* genes in liver. This induction was dependent upon the constitutive active/androstane receptor (CAR) because no induction occurred in *hUGT1Car^−/−^* mice. Induction of the *UGT1A* genes by TCC corresponded with induction of *Cyp2b10*, another CAR target gene. TCC was demonstrated to be a phenobarbital-like activator of CAR in receptor-based assays. While it has been suggested that TCC be classified as an endocrine disruptor, it activates ERα leading to induction of *Cyp1b1* in female ovaries as well as in promoter activity. Activation of ERα by TCC in receptor-based assays also promotes induction of human *CYP2B6*. These observations demonstrate that TCC activates nuclear xenobiotic receptors CAR and ERα both *in vivo* and *in vitro* and might have the potential to alter normal physiological homeostasis. Activation of these xenobiotic-sensing receptors amplifies gene expression profiles that might represent a mechanistic base for potential human health effects from exposure to TCC.

## Introduction

Triclocarban (3,4,4′-trichlorocarbanilide, TCC), containing the diphenyl urea moiety, is generally classified as a halogenated aromatic hydrocarbon. Along with triclosan [5-chloro-2-(2,4-dichlorophenoxy)phenol; TCS], TCC is used as a broad based antimicrobial agent [1] that is commonly added to personal hygiene products such as soaps and deodorants [2]. It has been estimated that the antibacterial agent is present in 76% of all liquid soaps, while TCC is the predominant antibacterial compound in all soaps [3]. Up to 454,000 kg of TCC is used in the United States each year [4]. As a result of its use in the health care industry and its resistance to degradation in sewage treatment processes, TCC has become a significant waste product that is found in numerous environmental compartments, such as water resources, sewage and sludge [4]–[6]. Evidence indicates that TCC passes freely through wastewater treatment facilities into the effluent and accumulates in the processed sludge, which is often used as a soil amendment or fertilizer, thus allowing TCC to be reintroduced into the environment. TCC has been detected at microgram per liter levels in water compartments in the environment [7], and it is among the top 10 most commonly detected organic waste water compounds for frequency and concentration indicating extensive contamination of aquatic systems [8], [9]. Its ubiquitous distribution in the environment has led to identification of TCC in biological fluids both in wild animals and humans [10]. The use of TCC containing soap products has demonstrated that TCC is readily absorbed through the skin and can be detected in urine samples following a single shower [11].

With the tendency of bioaccumulation in aquatic organisms [12], TCC’s ubiquitous presence in the environment has raised public concern regarding its potentially harmful effects in humans. Recent studies using *in vitro* bioassay tools have shown that TCC exhibits an association with various nuclear and steroid hormone receptors [10]. For example, studies with cell culture bioassays have demonstrated that TCC can interfere with the Ah receptor ligand binding by acting as an antagonist, and TCC can synergistically enhance testosterone action through interaction with the androgen receptor [10], [13], [14]. TCC evokes a modest synergistic response with a cell-based estrogen receptor (ER)-mediated bioassay in ERα positive human ovarian cancer cells [10], but does not serve as a direct ligand. It has been suggested that TCC be classified as an endocrine disruptor since it was shown to synergize the enlargement of male sex accessory organs in mice [13]. More recently, TCC has been shown to inhibit soluble epoxide hydrolase (sEH), which could potentially lead to biological regulation of inflammation, pain and blood pressure [15]. These findings suggest that TCC has the potential to influence human health.

Xenobiotic nuclear receptors (XenoRs) belong to a superfamily of transcription factors that regulate genes involved in drug metabolism and hormone homeostasis. Members of the XenoR superfamily each contain a ligand-binding domain (LBD) and a DNA binding domain (DBD) that targets the receptors to specific DNA sequence in the regulatory regions of potential target genes [16]. Upon ligand binding, the receptors translocate from the cytoplasm to the nucleus and shift to a transcriptionally active state, where they heterodimerize with the retinoic acid receptor (RXR) and become functional transcription factors that activate their target genes [17], [18]. Considering their modulation by small lipophilic molecules, we monitor activities of XenoRs, including pregnane X receptor (PXR), constitutive active/androstane receptor (CAR), liver X receptor alpha (LXRα), farnesoid X receptor (FXR), vitamin D nuclear receptor (VDR), peroxisome proliferator-activated receptor (PPARα), PPARβ, PPARγ, ERα, and ERβ, in response to TCC treatment. The initial study was to screen this wide variety of XenoRs for interaction with TCC, thus providing the framework for understanding the underlying mechanism through which TCC modulates gene regulation. To examine if TCC displayed CAR or PXR ligand specificity, we elected to initially evaluate the impact of TCC treatment in humanized *UGT1* (*hUGT1*) mice [19]. The human *UDP-glucuronosyltransferase 1 (UGT1)* locus and the nine *UGT1A* genes are expressed as a transgenic gene construct [20] in a *Ugt1*-null background [21] and express each of the *UGT1A* genes in a tissue specific fashion that is comparable to their expression patterns in human tissues [22]–[25]. In liver, it has been demonstrated that activation of PXR [20] and CAR [26] leads to induction of each of the *UGT1A* genes. In addition, murine target genes activated by PXR, such as *Cyp3a11* and CAR which target induction of *Cyp2b10,* can also be evaluated. Although TCC was shown previously to have little ER agonist activity in receptor-based bioassay screens [10], we elected to examine the impact of TCC on *Cyp1b1* expression *in vivo* in ERα sensitive tissues. Combined with techniques employing reverse genetics in mice and mechanistic studies in tissue culture, these findings add additional support to the current body of literature that TCC is capable of altering programmed gene expression, which may ultimately impact human health.

## Materials and Methods

### Cells, Transfections, and Culture Conditions

a) XenoR screening assay: Nuclear receptors (i.e., PXR, CAR, ERα, LXRα, VDR, FXR, PPARα, PPARδ, PPARγ, GR, and RXR), expression plasmids, and the luciferase reporter constructs containing their corresponding response elements were generated as described previously [18], [27]–[32]. CV-1 cells were maintained in phenol-red free DMEM (Life Technologies) supplemented with 10% super-stripped FBS, seeded in 96-well plates, and transfected with expression plasmids to supply a specific nuclear receptor (i.e., PXR, CAR, LXRα, VDR, FXR, PPARα, PPARδ, PPARγ, or GR) and RXR along with a luciferase reporter containing the appropriate DNA response element. The assay was conducted with TCC (10 µm) and various ligands as positive controls including pregnenolone 16α-carbonitrile (PCN, 10 µM; Sigma) for PXR, 1,4-Bis-[2-(3,5-dichloropyridyloxy)]benzene (TCPOBOP, 250 nM; Sigma) for CAR, β-estradiol (10 nM; Sigma) for ER, T0901317 (1 µM; Cayman Chemical) for LXRα, calcipotriol (10 µM; Sigma) for VDR, GW4064 (1 µM; Sigma) for FXR, WY14643 (30 µM; Sigma) for PPARα, GW501516 (100 nM) for PPARδ, rosiglitazone (1 µM; Cayman Chemical) for PPARγ, dexamethasone (100 nM; Sigma) for GR to validate the process of screening. b) ERα-mediated promoter activation: CV-1 cells were transfected with luciferase vectors containing the 2-kb promoter regions upstream of the start codon for *CYP1B1* or *CYP2B6* genes (Switch Genomics, CA) and the pcDNA3.1 expression vector alone or pcDNA3.1 expression vector for ERα. c) CAR ligand binding assays: CV-1 cells were transiently transfected with the expression vector containing the Gal4 DNA binding domain fused with the ligand binding domain of murine or human CAR, and co-transfected with the luciferase reporter plasmid, mh 100-luc [16]. All the transfection experiments were carried out using transient transfection reagent FugeneHD (Life Technologies) and transfection efficiency was measured by co-transfection of a β-gal expression vector. The day after the transfection, fresh phenol-red free medium with super-stripped serum containing DMSO, the positive compound of each nuclear receptor, or TCC (10 µM) was added, and the cells were incubated for an additional 24 hours. The luciferase activities were measured and normalized by β-gal activity [33]. The human breast adenocarcinoma cell lines MCF-7 and MDA-MB-231 were cultured in DMEM (4.5 mg/L glucose) supplemented with 10% fetal bovine serum (Invitrogen). Following TCC treatments, cells were incubated for 24 hours and harvested for total RNA isolation (Trizol, Invitrogen). Cell lines CV-1, MCF7, and MDA-231 were purchased from American Type Culture Collection (Manassas, VA).

### Animals

Humanized *UGT1* mice (hUGT1*28) were generated as described previously [19]. The *Car*-null (*Car ^−/−^*) mouse line was a generous gift from Dr. Negishi (National Institute of Environmental Health Sciences, Research Triangle Park, NC). Genotyping for *Car*-null mice was described previously [34]. Humanized UGT1*28 mice [19] were bred with *Car^−/−^* mice, and hUGT1*28/Car^+/−^ were backcrossed to produce hUGT1*28/Car^−/−^ mice. ***Ethics Statement:*** All animals received food and water *ad libitum,* and mouse handling and experimental procedures were conducted in accordance with our protocol (approval ID: No. S99100), previously approved by the University of California San Diego (UCSD) Institutional Animal Care and Use Committee (IACUC). The IACUC oversees the UCSD animal care and use program and is responsible for reviewing all animal use protocols, ensuring compliance with the federal regulations, inspecting animal facilities and laboratories and overseeing training and educational programs.

### In vivo Studies with hUGT1*28 and Car-null Mice

Age-matched groups of 6–8 week old animals were used, and mice were treated intraperitoneally (i.p.) every 24 hours for 2 days with Dimethyl Sulfoxide (DMSO) or TCC (16 mg/kg) dissolved in 50 µl DMSO for each injection. After 48 hours, the liver tissues, from each treatment group, were pulverized and used for preparation of total RNA. For experiments to detect *Cyp1b1* and *Cyp2b10* gene products in mouse ovary tissues, 10-day old female mice were treated with TCC (20 mg/kg) or corn oil by i.p. injection every other day for three weeks. Following the treatment, ovary tissues were used to prepare total RNA.

### Transfection of ERα-target Specific siRNA

Small interfering RNA (siRNA) duplexes were prepared by Bioneer (Alameda, CA). Targeted coding regions of the ERα oligonucleotides sequences were as follows iERα1, 5′- CTGTCTTCTGTTGGGAACA -3′, iERα2, 5′-GTCACTACTCAGGCTGACT -3′, and iERα3, 5′- CACTGAAATGGCCATTGAT -3′. MCF7 cells were seeded in a 6-well plate and transfected in the presence of 10 nM of either siRNA or negative-control RNA in a final volume of 1 ml OPTI-MEM with Lipofectamine 2000 (Invitrogen). After 5 hours, cells were replenished with fresh medium containing 10% fetal bovine serum. The next day after the transfection, cells were treated with either TCC or DMSO and incubated for an additional 24 hours followed by total RNA extraction. Real time RT-PCR results confirmed that transfection with iERαs reduced mRNA levels of ERα.

### Real-time Reverse Transcription-PCR

Mouse CYP2B10, human UGT1A1, 1A3, 1A4, 1A6 and 1A9, human CYP1B1 and human CYP2B6 mRNA levels were quantitated by real-time RT-PCR. Total RNA was isolated and cDNA was synthesized as described previously [35]. Following the cDNA synthesis, real-time PCRs were conducted with a pair of gene-specific primers and 2X MESA GREEN qPCR MasterMix (Eurogentec, San Diego, CA) to determine a Ct value of the corresponding gene using the MX4000 Multiplex Quantitative PCR (Stratagene, La Jolla, CA). Briefly, one micro liter of the cDNA template from the RT-PCR reaction was used in a 20 µl of reaction mixture containing 10 µl of 2X MESA Green qPCR MasterMix and 0.4 µM of a pair of corresponding primers. The forward and reverse primers for human CYP1B1 are (forward, 5′- TGACTGCCGTGTGTTTCGG -3′, and reverse, 5′-GTGCCTCAAGAACTTGTCCAG -3′); for human CYP2B6 the primers are (forward, 5′- AGACGCCTTCAATCCTGACC, and reverse, 5′- CCTTCACCAAGACAAATCCGC -3′), and those for mouse *Cyp2b10* and human *UGT* genes were described previously [20]. Each sample was performed in triplicate, normalized to the internal control genes mouse cyclophilin (forward, 5′- CAGACGCCACTGTCGCTTT -3′, reverse, 5′- TGTCTTTGGAACTTTGTCTGCAA) or human β-actin (forward, 5′- GGCGGCACCACCATGTACCCT -3′, and reverse, 5′- AGGGGCCGGACTCGTCATACT -3′), and quantified based on the formula ΔCt  =  Ct^(tested gene)^ – Ct^(cyclophilin)^.

### Microsomal Protein Preparation and Immunoblot Analysis

Age-matched mice were treated with either DMSO or TCC by i.p. injection for 48 hours. Following exposure by chemical treatment, mice were sacrificed and the microsomal fraction from mouse liver tissues was prepared as described previously [36]. The extracted microsomal protein (30 µg) was loaded on pre-cast Bis-Tris gel (NuPAGE, Novex) and electrophoresis was performed following determination of protein concentrations. The resolved protein was transferred onto a nitrocellulose membrane, and the membrane blocking, incubation with the primary antibody CYP2B10 (a kind gift from Dr. Negishi, NIEHS) and horseradish peroxidase-conjugated secondary antibody (Cell Signaling) were described previously [36]. Protein was detected by Renaissance Western Blot chemiluminescence reagent (PerkinElmer Life Sciences) and visualized using Bio-Rad ChemiDoc imager.

### Reagents

TCC, 1,4-bis[2-(3,5-dichloropyridyloxy)]benzene (TCPOBOP), and DMSO were from Sigma-Aldrich. (6-(4-Chlorophenyl)imidazo[2,1-*b*] [1], [3]thiazole-5-carbaldehyde-*O*-(3,4-dichlorobenzyl)oxime) (CITCO) was from Tocris Bioscience (St. Louis, USA). The Bradford assay for protein concentration analysis was from Bio-Rad Laboratories. The dual-luciferase reporter assay system and reporter plasmid, pGL3-basic vector and pRL-SV40 vector were from Promega. The plasmids for CAR ligand binding assay and CAR-mediated promoter activation was described previously [37]. The expression vector for ERα (pcDNA-ERα) was constructed in house (Evans Lab).

### Statistics

The values in the table and figures correspond to the mean ± standard deviation (SD) of at least three samples. Student’s t-test was used to assess differences between groups. Statistically significant differences are indicated with *, p<0.05; **, p<0.005; ***, p<0.0005.

## Results

### TCC Activates CAR and ERα

**Figure 1:**
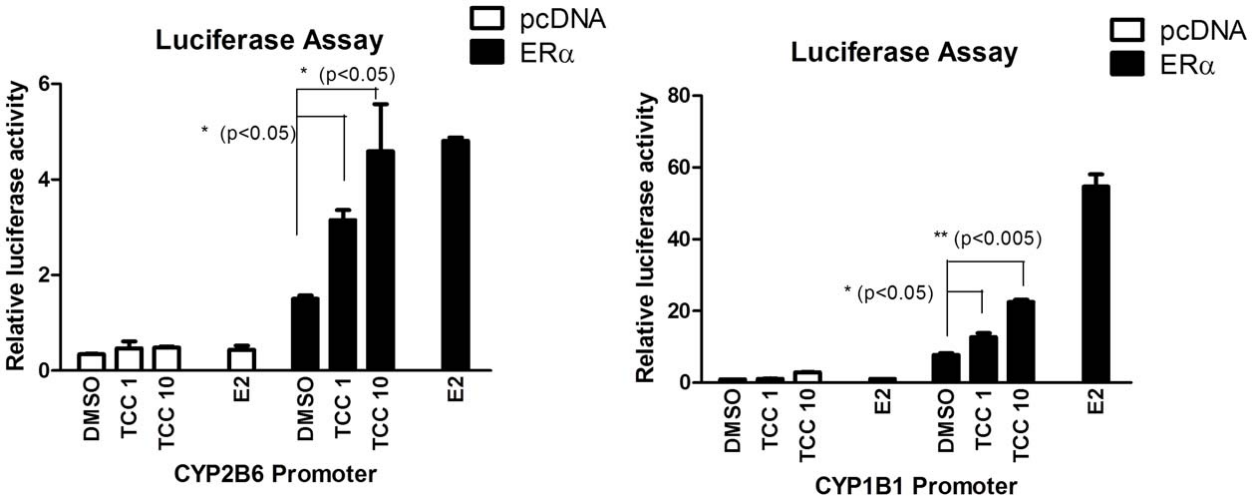
Promoter Activation by TCC. Transfection of a Luc reporter containing the 2 kb *CYP2B6* (A) or *CYP1B1* (B) promoter region with or without cotransfection of an ERα expression vector to CV-1 cells was performed. Following transfection, cells were treated for 24 hours with DMSO or different concentrations of TCC as indicated. Firefly luciferase activity was measured and normalized by using the level of β-galactosidase activity.

**Figure 2:**
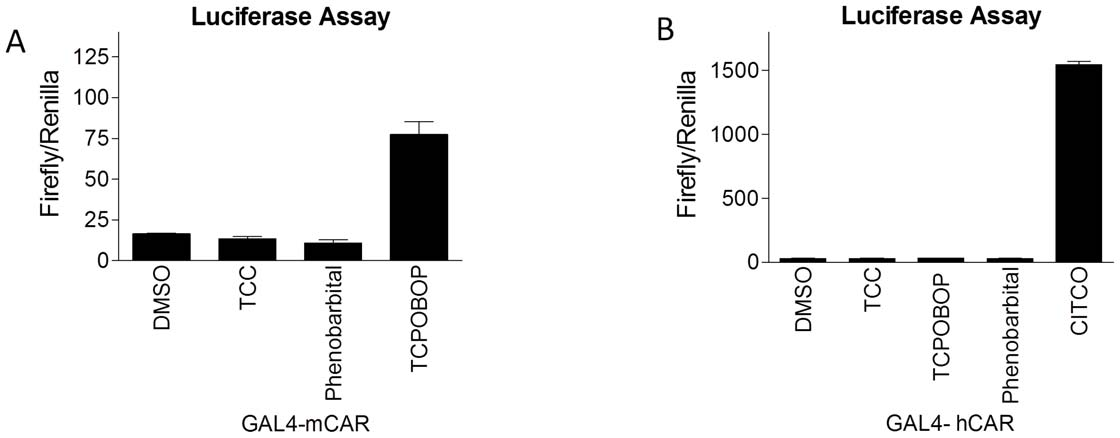
CAR ligand binding assay. HepG2 cells were transfected with the expression vector containing the Gal4 DNA binding domain fused with the ligand binding domain of murine CAR (mCAR, A) or human CAR (hCAR, B), and cotransfected with the Luciferase reporter plasmid, mh 100-luc. Following transfection, cells were treated for 24 hours with phenobarbital (1 mM), TCC (10 µM), TCPOBOP (25 µM), or CITGO (10 µM). Firefly Luciferase activity was measured and normalized by using the level of *renilla* luciferase activity.

***a) XenoR screening:*** Of the 11 XenoRs screened with TCC at the concentration of 10 µM, ERα and CAR were effectively activated by TCC (Table 1). CAR was moderately activated by TCC with 1.75-fold induction of luciferase activity. TCC also promotes ERα activity with similar potency as estradiol (Table 1). All other nuclear receptors produce statistically insignificant induction with VDR, FXR, PPARγ, and GR being mostly unaffected, thus TCC likely only has minimal effects on these receptors. ***b) ERα-mediated promoter activation:*** It has been shown previously that the human *CYP1B1* gene is regulated by estradiol through the ERα [38]. The ERα receptor has also been reported to regulate the expression of the human *CYP2B6* gene [39]. To identify the potential role of ERα in gene induction for *CYP1B1* and *CYP2B6* by exposure to TCC, transient transfections with a luciferase vector containing the 2kb fragment upstream from the *CYP2B6* start site (pGL3–2B6) with or without co-transfection of ERα were performed. The reporter activity of pGL3–2B6 increased with TCC treatment in a dose-dependent manner to a level compatible to that of estradiol treatment in the presence of ERα (Figure 1A). To further support the role of ERα in TCC-mediated gene regulation, gene expression of human *CYP1B1*, a well-characterized enzyme that is linked to expression in estrogen-regulated tissue through ERα [38] was examined. Similar to the *CYP2B6* response to TCC, *CYP1B1* promoter activity was significantly induced by TCC in a dose-dependent fashion. Mimicking the estradiol action, ERα is required for the *CYP1B1* promoter activation by TCC (Figure 1B). ***c) CAR ligand binding assay***: CAR has been shown to display a species divergence in ligand specificity. Human CAR (hCAR), but not murine CAR (mCAR), is strongly activated by CITCO, but the mouse receptor is more sensitive to TCPOBOP than hCAR [40]. We further conducted transfection experiments using a fusion protein containing the mCAR ligand-binding domain and the Gal4 DNA-binding domain along with a Gal4 responsive luciferase reporter, mh100, showed no change in luciferase expression levels to TCC, while TCPOBOP elicited a robust induction as a positive control. Similarly, hCAR exhibited no response to TCC with CITCO as a potent ligand (Figure 2A and 2B) suggesting that TCC is a CAR activator, but not an agonist ligand for either mouse or human CAR.

**Table 1:** Xenobiotic receptors screening assay.

Xenobiotic receptors	Reporter constructs	Fold induction in the absence of ligand	Fold induction with the positive ligand	Fold induction with TCC
Erα	ERE	1±0.1	2.89±0.26	2.13±0.14**
Erβ	ERE	1±0.06	1.85±0.26	1.47±0.15
CAR	PBRE	1±0.07	2.05±0.33	1.75±0.30***
PXR	CYP3A promoter	1±0.27	4.22±0.73	1.44±0.44
LXRα	LXRE	1±0.4	2.34±1.49	1.51±0.73
VDR	CYP24A1 promoter	1±0.45	9.67±3.94	1.06±0.07
FXR	FXRE	1±0.14	29.3±3.79	0.88±0.18
PPARα	PPRE	1±0.13	8.6±2.89	1.53±0.27
PPARδ	PPRE	1±0.32	10.36±0.64	1.57±0.55
PPARγ	PPRE	1±0.32	5.89±0.64	1.03±0.55
GR	GRE	1±0.1	5.83±2.17	1.1±0.18

TCC was tested for the ability to activate PXR, CAR, LXRα, FXR, VDR, PPARα, PPARβ, PPARγ, ERα, and ERβ by binding to the corresponding response element. CV1 cells were transfected with an expression plasmid to supply a human nuclear receptor, a luciferase reporter plasmid containing the appropriate DNA response element, and a β-galactosidase expression vector to control for transfection efficiency. The positive ligand for each receptor is described in Material and Method. Luciferase activity was represented as fold induction relative to DMSO-treated cells. The results are expressed as the mean ± S.D.

** p<0.005.

*** p<0.0005, significant difference between the values for DMSO- and TCC-treated wells.

### TCC Induction of Xenobiotic Metabolism by CAR

**Figure 3:**
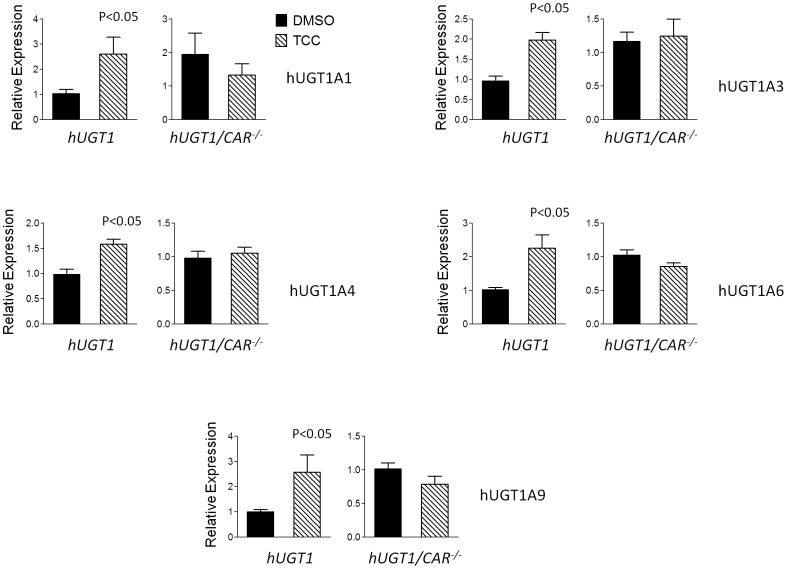
The comparative expression of hepatic UGT1A genes between hUGT1 and hUGT1/Car^−/−^ mice. RT-PCR analysis using liver RNA from DMSO- or TCC-treated (16 mg/kg, dissolved in 50 µl DMSO) mice and isoform specific-primer pairs was performed to detect the expression of *UGT1A* genes as indicated.

**Figure 4:**
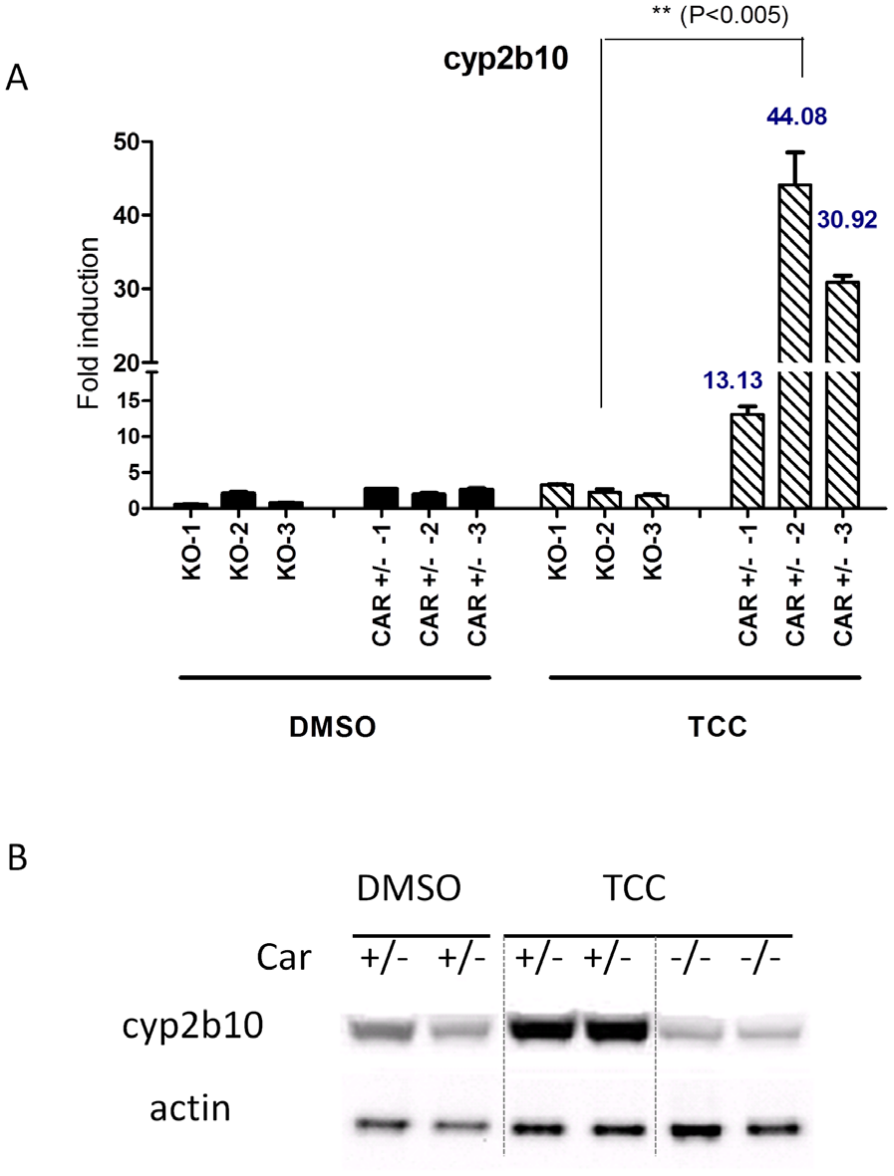
Cyp2b10 gene expression following the TCC treatment. Age-matched heterozygous (*CAR ^+/−^*) and *Car*-null mice (*Car ^−/−^*) (n = 3) were treated with either DMSO or TCC (16 mg/kg) by intraperitoneal injection for 48 hours. A. The liver tissues were used for preparation of total RNA. After the reverse transcription for cDNA synthesis, real-time PCR was conducted to determine the Ct value with cyclophilin as an internal control gene. B. The microsomal proteins were prepared from liver tissues and subject to Western Blot analysis using Cyp2b10 antibody as the primary antibody.

The *UGT1A* genes expressed in hUGT1*28 mice are susceptible to regulation by CAR [26], PXR [20], peroxisome proliferator-activated receptor-alpha (PPARα) [41] and the Ah receptor [20]. Initial experiments were conducted by treating hUGT1*28 mice with 16 mg/kg TCC by the i.p. route and evaluating *UGT1A* gene expression patterns in liver after 48 hours. In comparison to DMSO treated mice, *UGT1A1*, *−1A3*, *−1A4*, *−1A6*, and *−1A9* gene products were each induced in hUGT1*28 mice following TCC administration (Figure 3). When gene targets that are regulated by CAR (*Cyp2b10*), PXR (*Cyp3a11*), PPARα (*Cyp4a11*) and the Ah receptor (*Cyp1a1*) were evaluated by real time RT-PCR analysis, only *Cyp2b10* gene expression was found to be induced (Figure 4), indicating that CAR activation by TCC was leading to the induction of the *UGT1A* genes. To examine if CAR was underlying the induction pattern, we treated hUGT1*28/Car^−/−^ mice with TCC and examined expression of the *UGT1A* genes. In comparison to hUGT1*28 mice, there was no induction of the *UGT1A* genes in hUGT1*28/Car^−/−^ mice. This correlated with a nearly complete absence of *Cyp2b10* gene induction following TCC treatment in hUGT1*28/Car^−/−^ mice (Figure 4A). To access the expression of Cyp2b10 protein in microsomal preparation from treated mouse livers, Western blot analysis was performed with antibody against the CYP2B6 protein. Consistent with the transcript levels, Cyp2b10 protein expression was induced by TCC treatment, and the induction was blocked in Car^−/−^ livers (Figure 4B).

### CYP2B6 and CYP1B1 Gene Expression Regulated by TCC

**Figure 5:**
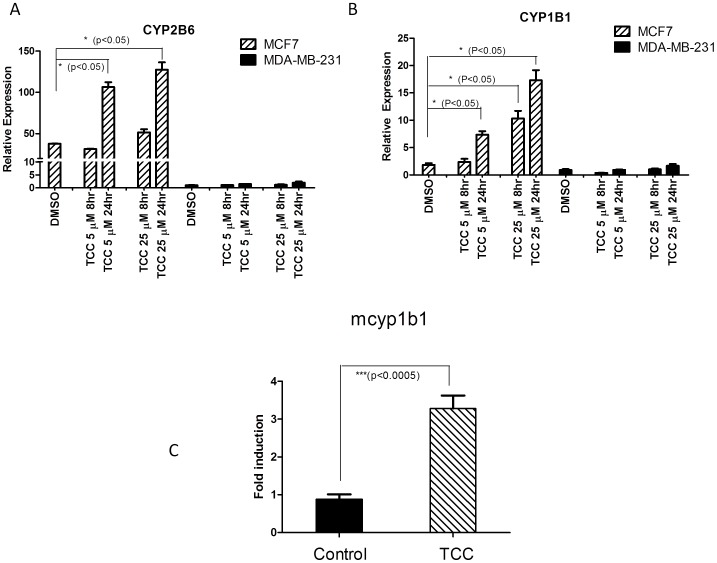
Activation of gene expression by TCC. Human breast cancer cell lines, MCF7 and MDA-MB-231, were cultured and treated with either DMSO or 5 or 10 µM TCC. Following total RNA isolation and reverse transcription reaction, *CYP2B6* (A) or *CYP1B1* (B) mRNA levels were measured by real-time PCR using a gene specific pair of primers. (C) Induction of cyp1b1 gene in mouse ovary and fallopian tube by TCC. 10-day old hUGT1*28 mice were treated with corn oil or TCC (20 mg/kg) by i.p. injection every 48 hours until 30 days of age. The tissues of ovaries and fallopian tubes were used to prepare total RNA. Following the reverse transcription for cDNA synthesis, real-time PCR was conducted to determine the Ct value for *cyp1b1* with cyclophilin as an internal control gene.

**Figure 6:**
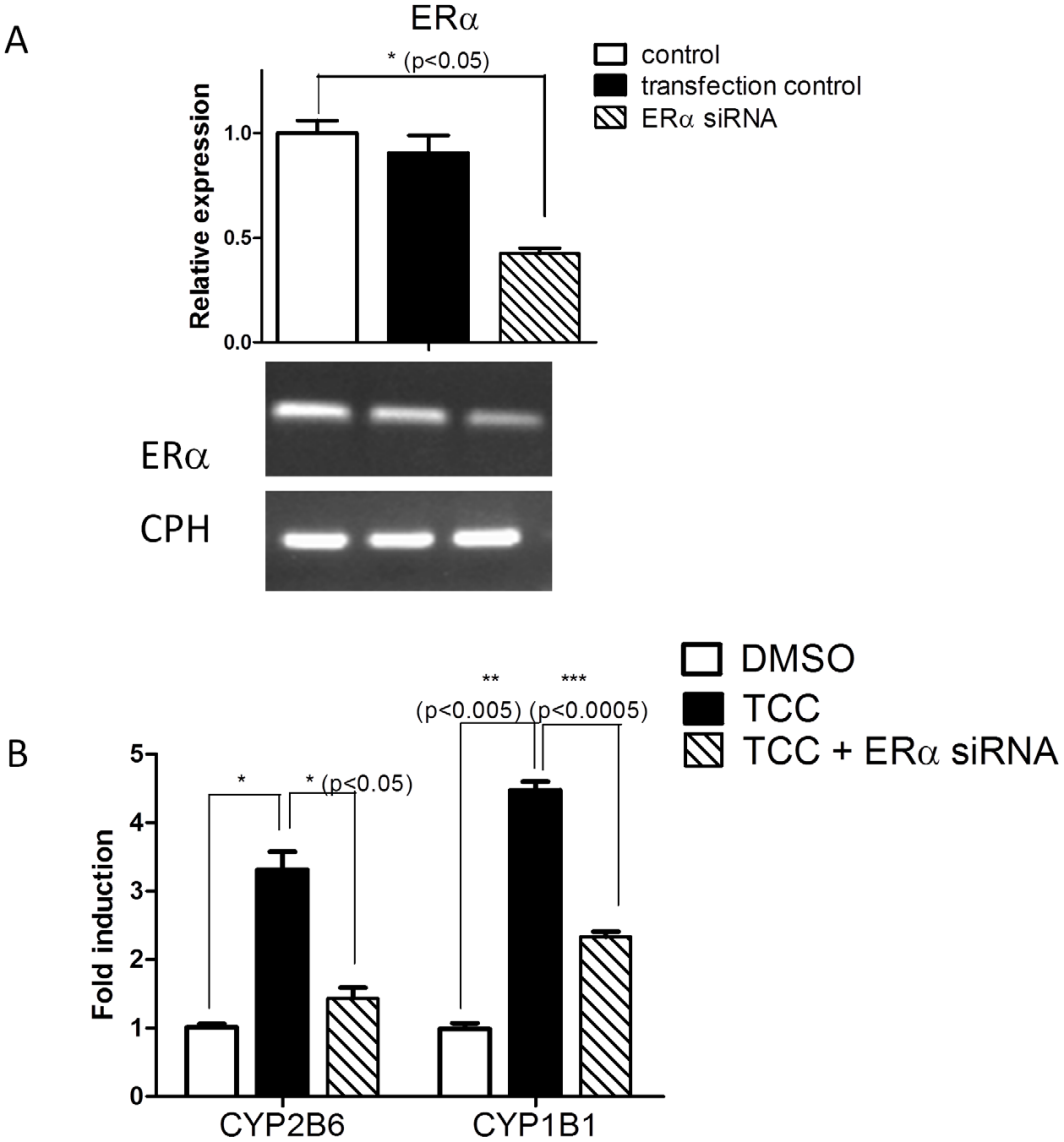
TCC-mediated gene induction is blocked by ERα siRNAs. siRNAs for ERα were designed and synthesized by Bioneer (Alameda, CA) and dissolved in DEPC-H_2_O. MCF7 cells were seeded in a 6-well plate and transfected with either ERα siRNAs or random siRNAs (transfection control) using lipofectamine 2000. The next day, cells were treated with either TCC or DMSO and incubated with either TCC (25 µM) or DMSO for 24 hours. Following treatments, total RNA was extracted. (A) ERα mRNA levels were evaluated by real time and RT-PCR with cyclophilin (CPH) as an internal control gene. (B) CYP2B6, and CYP1B1 mRNA levels were measured by real time PCR with CPH as an internal control gene and displayed as fold induction compared with DMSO-treated samples as one fold.

To investigate the effect of TCC on CYP2B6 mRNA expression through a ERα-dependent mechanism, ERα positive MCF7 and ERα negative MDA-MB-231 human breast cancer cells were treated with TCC. Results of real time RT-PCR revealed dose- and time-dependent effects of TCC treatment on induction of CYP2B6 mRNA expression in ERα positive MCF7 cells (Figure 5A). In contrast, *CYP2B6* expression was unaffected in TCC-treated ERα negative MDA-MB-231 cells. To further support the role of ERα in TCC-mediated gene regulation, gene expression of human *CYP1B1*, a well-characterized enzyme that is linked to expression in estrogen-regulated tissue through ERα [38] was examined. Similar to *CYP2B6* response to TCC, MCF7 cells exhibited a TCC-dependent increase in CYP1B1 mRNA levels that was not observed in MDA-MB-231 cells, further assuring the involvement of ERα in TCC-mediated gene induction (Figure 5B). Furthermore, we examined mouse *Cyp1b1* induction in ERα sensitive tissue such as the ovaries. TCC treatment to female hUGT1*28 mice resulted in the induction of *Cyp1b1* (Figure 5C). These results clearly link TCC exposure to activation of ERα target genes, *Cyp1b1/CYP1B1* and *Cyp2b10/CYP2B6* in both cultured human breast cancer cells and *in vivo* estrogen-sensitive tissues. Finally, transfection experiments with siRNAs targeting ERα were performed to interrupt ERα gene expression in MCF7 cells. Gene expression levels for *CYP1B1*, *CYP2B6*, and ERα were analyzed by real time PCR following siRNA transfections and TCC treatment. SiRNA knockdown of ERα with 58% effectiveness significantly reduced *CYP1B1* and *CYP2B6* gene expression in MCF7 cells (Figure 6), further assuring the involvement of ERα in TCC-mediated gene induction.

## Discussion

With the XenoR screening assay, our results show divergent receptor binding activities of TCC as indicated by fold induction of luciferase activity. This system appears to be a suitable qualitative model for evaluating XenoR activation toward tested compounds. CAR and ERα appeared most susceptible to TCC activation and exhibited moderate luciferase activity. We then assessed potential biological effects arising from TCC exposure in the perspective of drug and steroid metabolism and discussed the molecular mechanism through which the key phase I and phase II xenobiotic metabolizing enzymes are induced. The use of *in vitro* cell-based assays to predict and define the underlying mechanisms combined with TCC exposure within *in vivo* animal models is a useful approach to understand xenobiotic interactions caused by TCC. An indirect interaction of TCC with CAR that enables TCC to induce the human *UGT1A* genes in hUGT1*28 mice was revealed in our animal models. The specificity of CAR-mediated regulation by TCC is supported by observations that hepatic *Cyp2b10* is also induced, while the *UGT1A* genes and the *Cyp2b10* gene are not induced in hUGT1*28/Car^−/−^ mice. We also eliminated any interaction with PXR, since PXR was insensitive to TCC in the luciferase reporter assay, and TCC has no effect on expression of the PXR-target gene *Cyp3a11* (data not shown). CAR is well recognized to regulate the induction of the *CYP2B* genes by PB and a group of diverse agents referred to as “PB-like” agents including the pesticide contaminant TCPOBOP, the most potent PB-like inducers known in rodents [42]. To date, only a few CAR agonists, such as TCPOBOP for murine CAR and CITCO for human CAR are identified. Our results with the GAL4-human/mouse CAR LBD fusion proteins, representing the ligand specificity of CAR, indicate that TCC, mimicking PB, acts as an indirect CAR activator. Although the luciferase activation by TCC was at a moderate level, this induction was significant compared to that of the control and 2-fold induction by TCPOBOP. The moderate scale CAR activation in the luciferase screening assay by TCC may be due to the unique feature of CAR being a constitutive active receptor even in the absence of an exogenous ligand as well as being a CAR activator through an indirect mechanism that facilitates translocation of CAR to the nucleus and transactivation of its target genes.

The ability of TCC to stimulate CAR target genes may be of significance. The current study has demonstrated cross-regulation with *CYP2B* and human *UGT1A* genes by TCC through CAR activation. This is not surprising because a PB response element flanking the gene promoter has been identified in both human *CYP2B6* and *UGT1A1* genes [37], [39]. It is well characterized that the CAR-mediated inducible expression of xenobiotic-metabolizing enzymes and accelerated metabolic elimination of corresponding substrates is associated with drug-drug interaction clinically. In addition, CAR can act as a negative regulator controlling inhibition of biotransformation genes, e.g., *UGT2B7* and *CYP7A1* [35], [43]. This down regulation might lead to changes in steady-state dynamics of steroids and bile acid homeostasis. Thus, TCC contamination in biological systems may be critically relevant by interfering with metabolism of endogenous and exogenous substrates by CAR-target xenobiotic genes. For example, a recent study has linked CAR pre-activation and alcohol infusion to a synergistic decrease in the expression of enzymes that metabolize the alcohol in liver including alcohol dehydrogenase 1, aldehyde dehyrogenase (ALDH) 1A1, ALDH3A2, and CYP2E1, which supports the role of CAR in modulating alcoholic liver injury [44]. Secondly, it has been characterized that PB-like agents possess CAR-dependent liver enlargement properties that are evidenced by increases in cell proliferation and suppression of apoptosis [45]. This CAR-dependent action is thought to be a prerequisite and underlying mechanism for nongenotoxic PB-mediated liver tumorigenesis [46]. Therefore, TCC might deserve close scrutiny to determine if its long-term effects on liver disease and tumorigenesis through a CAR dependent mechanism, similar to the actions of PB.

While transcriptional regulation of the *CYP2B* gene is evident through TCC-mediated CAR activation, the slight induction of *Cyp2b10* in TCC-treated CAR null livers (1.77–3.26 fold induction compared with DMSO-treated ones) implies the CAR-independent mechanism. Recently, a study has confirmed ERα-dependent regulation of *CYP2B6*, in which a functional ER response element was identified in the *CYP2B6* regulatory region [39]. In this study, we provide compelling evidence showing CYP2B6 induction by TCC is also mediated through ERα. In addition to CAR, TCC enables induction of *CYP2B6* promoter activity in an ERα-dependent fashion. Moreover, ERα activation by TCC affects other ERα target genes; *CYP1B1* transcription is subjected to ERα-dependent TCC regulation as evidenced by activation of the *CYP1B1* promoter region, which was previously characterized as harboring a functional ERα binding site [38]. TCC-mediated induction of *CYP2B6* and *CYP1B1* was observed in estrogen sensitive cultured cells and tissues further supporting the observation that TCC exposure can regulate target genes of ERα through an ERα-dependent mechanism.

Human TCC metabolism involves direct N− and N′- glucuronidation and ring hydroxylation to 2-OH-TCC and 6-OH-TCC, which can further undergo sulfate and glucuronide conjugation [47]. TCC metabolites are detected in plasma and urine in subjects who have showered with TCC-containing soap with TCC-N-glucuronides as the major route of renal excretion [11]. The robust TCC-mediated *CYP2B* gene induction through both CAR and ERα activation may be relevant considering that contact to TCC maybe occurring consistently over time through the use of personal care products. CYP2B6 is one of the major enzymes to metabolize clinical drugs (e.g., cyclophosphamide and tamoxifen), and 20− to 250-fold inter-individual variation in CYP2B6 expression has been demonstrated [48], presumably due to transcriptional regulation and polymorphisms. Environmental exposure to agents like TCC may be contributing towards variation in *CYP2B6* since both CAR and ERα are regulators of this gene and potential targets following TCC exposure.

With environmentally relevant concentrations, a recent report indicated that TCC increased embryo production in the freshwater mud snail suggesting that TCC may be causing reproductive effects in a manner similar to that seen with some known environmental estrogens [49]. Although TCC is not an agonist for the AR (androgen receptor), TCC has the ability to amplify the effect of testosterone on the androgenic activity in the AR-mediated luciferase assay [13]. To study ER interactions, Ahn et al. [10], [14] performed a cell-based ER-mediated bioassay to study TCC-ERα interaction. At 1 and 10 µM, TCC evoked a modest response equivalent to about 30% of that produced by 1 nM estradiol. Although it is a mild agonist for the ERα in the reporter gene assay, TCC enhances the estrogen action in the presence of estrogen [10]. Combined with our results, it can be anticipated that TCC has a significant influence on modulating transcriptional control of ERα target genes, such as those involved in xenobiotic and steroid metabolism. For example, regulating *CYP1B1* gene expression by TCC reinforces its ability to disrupt estrogen homeostasis. Estrogen has long been associated with breast cancer. CYP1B1 is highly expressed in estrogen target tissues and changes in the expression of proteins such as CYP1B1 are known to metabolize estrogens, which can potentially alter physiological levels and the intensity of estrogen action. In addition, CYP1B1 catalyzes the 4-hydroxylation of estradiol to generate 4-hydroxy estradiol [50], a catechol metabolite that produces free radicals, causes cellular damage, and is associated with carcinogenic activity of estrogen and the development of breast cancer. The continual use of personal care products that contain antimicrobial agents like TCC may enhance over time the generation of endogenously produced metabolites capable of eliciting a toxic or carcinogenic episode. In addition, CAR activation has recently been shown to inhibit human *UGT2B7* gene expression [35]; indicating that continued exposure to TCC would have a negative influence on normal drug metabolism and clearance. Combined with evidence that there is considerable cross-talk between the ER and CAR [51], [52], TCC accumulation may surely influence the many target genes involved in xenobiotic metabolism. Therefore, the perceived benefits of using TCC as an antimicrobial agent in personal care products should be weighed against possible risks.

### In Conclusion

These studies demonstrate that acute exposure to TCC results in the activation of important regulatory pathways dictated by CAR and ERα that can potentially impact the steady-state levels of hormones, as well as altering routes of drug metabolism. One of the important check points in steroid homeostasis is biological inactivation by glucuronidation, which is shown in this study to be induced in a CAR dependent fashion following TCC treatment. Induction of cytochrome P450 genes, whose products are steroid hydroxylases, is also regulated by TCC both through CAR as well as ERα. Combined with findings that TCC is capable of synergizing the actions of testosterone through the AR [13], these studies suggest that long term exposure to TCC, from daily use of personal hygiene products, has the potential of altering normal steroid biogenesis. Thus, long term alterations in hormone homeostasis initiated by chronic exposure to TCC could potentially lead to human health problems.
